# Supplementary material for: Room temperature chirality switching and detection in a helimagnetic MnAu2 thin film
Source: Nat Commun. 2024 Mar 7;15:1999. doi: 10.1038/s41467-024-46326-4 (PMC10920692; doi:10.1038/s41467-024-46326-4)
Supplement: Supplementary file 1 — Supplementary Information [file 41467_2024_46326_MOESM1_ESM.pdf]

Supplementary Information for

Room temperature chirality switching and detection in a helimagnetic $MnAu_2$ thin film

Hidetoshi Masuda*, Takeshi Seki*, Jun-ichiro Ohe, Yoichi Nii, Hiroto Masuda, Koki Takanashi, Yoshinori Onose*

*Correspondence Authors. Email: hidetoshi.masuda.c8@tohoku.ac.jp (H. M.); takeshi.seki@tohoku.ac.jp (T. S.); yoshinori.onose.b4@tohoku.ac.jp (Y. O.)

1. Numerical calculation of current-induced spin-polarization in a chirality-variable helimagnet with a centrosymmetric crystal structure

In the literature (ref. 1), the current-induced spin polarization was theoretically discussed for a helimagnet induced by the Dzyaloshinskii–Moriya interaction, in which the chirality is fixed. To confirm the validity for a chirality-variable system, we numerically investigate the spin polarization induced by an electric current in a conducting helimagnet with variable chirality. First, we obtain a stable magnetic helical structure in a variable-chirality helimagnetic model (classical J_1-J_2 Heisenberg model) by solving the Landau-Lifshitz-Gilbert (LLG) equation. Second, the spin polarization induced by an electric current is calculated by using Green's function method, considering the interaction between the spin conduction electron and the localized moment with the obtained magnetic structure[1].

The Hamiltonian of classical J_1-J_2 Heisenberg model is

$$\mathcal{H}_{\mathrm{mag}} = -\sum_{\langle i,j\rangle} J_1 \mathbf{S}_i \cdot \mathbf{S}_j - \sum_{\langle\langle i,j\rangle\rangle} J_2 \mathbf{S}_i \cdot \mathbf{S}_j + \sum_i K {S_{ix}}^2 \tag{1}$$

where the first sum $\langle i,j\rangle$ runs over the nearest neighbor and the second $\langle\langle i,j\rangle\rangle$ over the next nearest neighbor spin pairs. K is the anisotropic energy of the easy y-z plane. We use the parameters $J_1 = 10$ meV and $K = 10^{-2}$ meV. The system size is 100×30 spins with $S = 1.3\ \mu_{\mathrm{B}}$, and the lattice constant is $a = 5$ nm. To obtain the spin structure, we solve the LLG equation

$$\frac{\partial \mathbf{S}_i}{\partial t} = -\gamma \mathbf{S}_i \times \left(\mathbf{H}_{\mathrm{eff}\,i} + \mathbf{H}_{\mathrm{temp}}\right) - \frac{\alpha}{S}\left(\mathbf{S}_i \times \frac{\partial \mathbf{S}_i}{\partial t}\right) \tag{2}$$

where $\mathbf{H}_{\mathrm{eff}\,i} = -\partial\mathcal{H}_{\mathrm{mag}}/\partial\mathbf{S}_i$ is the effective magnetic field acting on the spin $\mathbf{S}_i$. $\mathbf{H}_{\mathrm{temp}}$ is the fluctuation field that introduces the finite temperature, which satisfies $\langle H^{\eta}_{\mathrm{temp}}(t) H^{\kappa}_{\mathrm{temp}}(t')\rangle = \frac{2\alpha k_{\mathrm{B}} T}{S}\delta_{\eta\kappa}\delta(t-t')$, where η and κ are Cartesian coordinates. Here we assumed $T = 10$ K. $\gamma = 1.75\times10^{10}$ T^{-1}s^{-1} is the gyromagnetic ratio. $\alpha = 0.05$ is the Gilbert damping coefficient. First, we assumed an initial magnetic structure as the helical state $\mathbf{S}_i = (0, \cos qx_i, \sin qx_i)$, where x_i is the x coordinate of the $\mathbf{S}_i$ position. q is the initial helimagnetic wave vector. To obtain the proper configuration of the spin system, we calculated the time evolution with the LLG equation by using the 4th-order Runge-Kutta method. Due to the Gilbert damping term, the spin system forms a minimum-energy state after sufficient time evolution. In the present system, the helical state is obtained when $J_1 > 0$ and $J_2 < 0$. The helical state is characterized by the helicity λ defined as

$$\lambda = \left(\mathbf{S} \times \frac{\partial \mathbf{S}}{\partial x}\right)_x . \tag{3}$$

$\lambda > 0$ ($\lambda < 0$) corresponds to the right (left) handed chirality. Because the spin interaction is symmetric, the chirality is determined by the initial state. In this case, the sign of q determines it. Note that the control of the chirality is achieved by applying the charge current[2,3].

The conduction electrons coupled to the magnetic structure are described by the s-d model,

$$\mathcal{H}_{\mathrm{el}} = -V_0 \sum_{\langle i,j\rangle,\sigma} c_{i\sigma}^{\dagger} c_{j\sigma} - J_{\mathrm{sd}} \sum_{i,\sigma,\sigma'} c_{i\sigma}^{\dagger} \hat{\boldsymbol{\sigma}}_{\sigma,\sigma'} c_{\sigma'} \cdot \mathbf{S}_i \tag{4}$$

where $c_{i\sigma}$ ($c_{i\sigma}^{\dagger}$) is the annihilation (creation) operator of an electon at the i site with the spin σ. $\mathbf{S}_i$ is the magnetization of the i site that is calculated by the LLG equation. V_0 ($= \hbar/2m^{*}a$) is the transfer energy of the conduction electrons. a is the lattice constant and $m^{*} = 0.067m_e$ is the effective mass. We set the conduction electron energy (E_F) to 0.5 V_0. The spin polarization of the conduction electrons in the v-direction is defined as

$$P_v = \frac{\mathrm{Tr}(\hat{t}^{+}\hat{\sigma}_v\hat{t})}{\mathrm{Tr}(\hat{t}^{+}\hat{t})} \tag{5}$$

where $\hat{t}$ is the transmission matrix represented in the 2×2 spin space and $\hat{\sigma}_v$ are the Pauli matrices). The transmission matrix is calculated by the Green's function method[4]. P_v measures the spin-polarization of electric current. Therefore, spin accumulation is induced under an electric current when P_v is finite. Supplementary Figure 1 shows the calculated helicity $\lambda = \left(\mathbf{S} \times \frac{\partial \mathrm{S}}{\partial x}\right)_x$ and spin polarization of conductance $P_x = \mathrm{Tr}(\hat{t}^{+}\hat{\sigma}_x\hat{t})/\mathrm{Tr}(\hat{t}^{+}\hat{t})$ as a function of $|J_2/J_1|$. Helicity λ emerges around $|J_2/J_1| = 0.05$ and increases rapidly around $|J_2/J_1| = 0.25$. Whereas the helimagnetic state is known to be stabilized for $|J_2/J_1| > 0.25$ in an infinite J_1-J_2 classical model[5], the ferromagnetic-helimagnetic transition seems to be broadened in this finite model. The chirality could be controlled by the suitable choice of initial states. Importantly, P_x that depends on the chirality begins to evolve around $|J_2/J_1| = 0.25$. Note that the spin polarization oscillation with the ratio $|J_2/J_1|$ seems to be caused by a resonance condition between the Fermi wavelength and the helical pitch. The calculation numerically demonstrates the electric current-induced spin polarization with a sign that depends on the chirality.

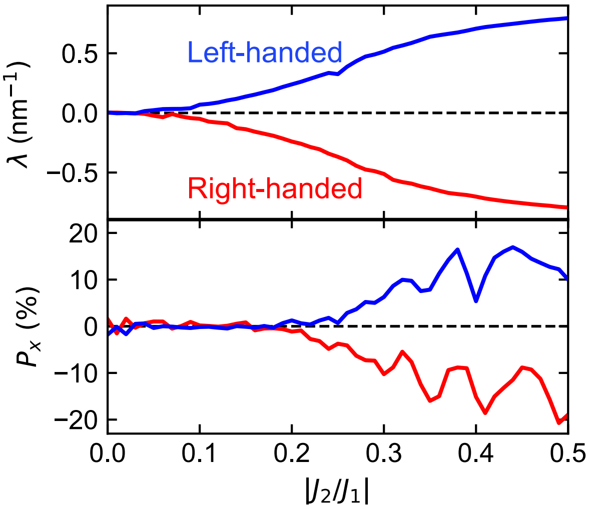

Supplementary Fig. 1 | Calculated helicity and spin polarization of conductance $\lambda = \left(\mathbf{S} \times \frac{\partial \mathbf{S}}{\partial x}\right)_x$ and P_x along the helimagnetic propagation vector as a function of $|J_2/J_1|$, where J_1 (J_2) is the ferromagnetic nearest-neighbor (antiferromagnetic next-nearest-neighbor) magnetic interaction of localized moments.

2. nonreciprocal electronic transport

This section illustrates why nonreciprocal electronic transport arises in helimagnetic $MnAu_2$ films. Nonreciprocal responses are uniaxial responses that arise when spatial inversion and time-reversal symmetries are simultaneously broken. In the case of electronic transport, Rikken and coworkers first showed the nonreciprocity[6]. For diagonal electronic conductivity σ, Onsager's relation suggests

$$\sigma(\mathbf{k}, \mathbf{H}) = \sigma(-\mathbf{k}, -\mathbf{H}), \tag{6}$$

where $\mathbf{k} = (k_x, k_y, k_z)$ and $\mathbf{H} = (H_x, H_y, H_z)$ are the wave vector and the magnetic field. Therefore, by the expansion, we get

$$\sigma = \sigma(0,0) + \sum \alpha_{ij} k_i H_j + \cdots \tag{7}$$

where α_{ij} is constant. α_{ij} is zero if the inversion symmetry is preserved but finite if broken. In isotropic chiral materials,

$$\sigma = \sigma(0,0) + \alpha \mathbf{k} \cdot \mathbf{H} + \cdots. \tag{8}$$

Rikken *et al.* suggested that the electric current I can substitute $\boldsymbol{k}$ in the expansion and deduced

$$\sigma = \sigma(0,0) + \alpha I H + \cdots. \tag{9}$$

Therefore, for chiral materials, $\sigma(+I) \neq \sigma(-I)$ in a magnetic field. This is the electronic version of nonreciprocal response. In this case, the resistance R and voltage V show the relation

$$R = R(0,0) + \beta IH + \cdots, \quad (10)$$
$$V = RI = R(0,0)I + \beta I^2 H + \cdots, \quad (11)$$

where β is constant. These relations indicate the second harmonic resistivity is finite and odd with respect to the magnetic field (or time reversal operation) in chiral materials. Because helimagnets are symmetrically classified into chiral materials, the nonreciprocal electronic resistivity is observed.

3. Numerical calculation for the magnetic anisotropy dependence of chirality switching

To discuss how to decrease the critical current of chirality switching and to extend the working temperature region, we did the numerical calculations similarly to Fig. 4 in the literature[3]. Helicity as a function of applied current density was numerically calculated with varying the magnitude of magnetic anisotropy (K) as shown in Supplementary Fig. 2, in which other conditions are the same as ref. 3. The critical current is decreased as the magnetic anisotropy is decreased. Therefore, the decrease of magnetic anisotropy is an effective way for decreasing the critical current and extending the working temperature.

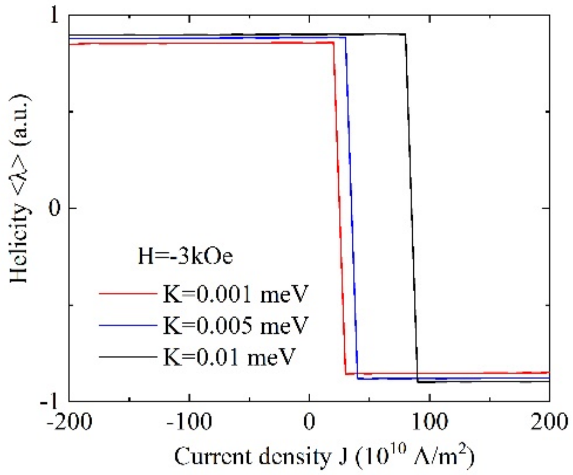

Supplementary Fig. 2 | Numerical calculation for the magnetic anisotropy dependence of chirality switching.

Numerical calculation for the chirality switching by using the charge current and the magnetic field with several magnitudes of magnetic anisotropy K. The initial state has positive helicity. The other conditions are the same as ref. 3.

4. Supplementary experimental data

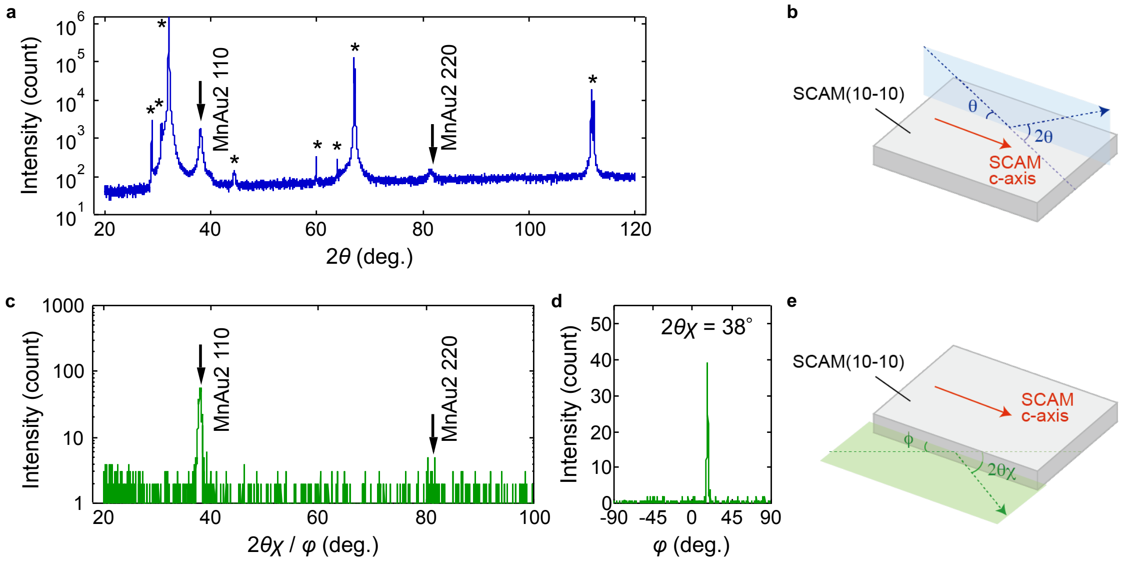

Supplementary Fig. 3 | XRD profiles of the $ScMgAlO_4$ (10−10) / $MnAu_2$ (110) [100nm] / Ta[2nm] thin film sample.

a Out-of-plane $\theta/2\theta$-scan. $MnAu_2$ (110) and (220) reflections indicate that the $MnAu_2$ (110) plane is grown on the $ScMgAlO_4$ (10−10) plane. Asterisks denote the peaks from the SCAM ($ScMgAlO_4$) substrate or sample stage. No extra reflections were observed.

b Schematic illustration of the out-of-plane $\theta/2\theta$-scan.

c In-plane $2\theta\chi/\varphi$-scan. The observation of $MnAu_2$ (110) and (220) reflections indicates that the $MnAu_2$ [1−10] direction is perpendicular to the $ScMgAlO_4$ [0001] direction (see Supplementary Fig. 3e).

d In-plane φ-scan. A single sharp peak in the φ-scan range from −90° to 90°indicates the in-plane single-crystal order.

e Schematic illustration of the in-plane $2\theta\chi/\varphi$- and φ- scans.

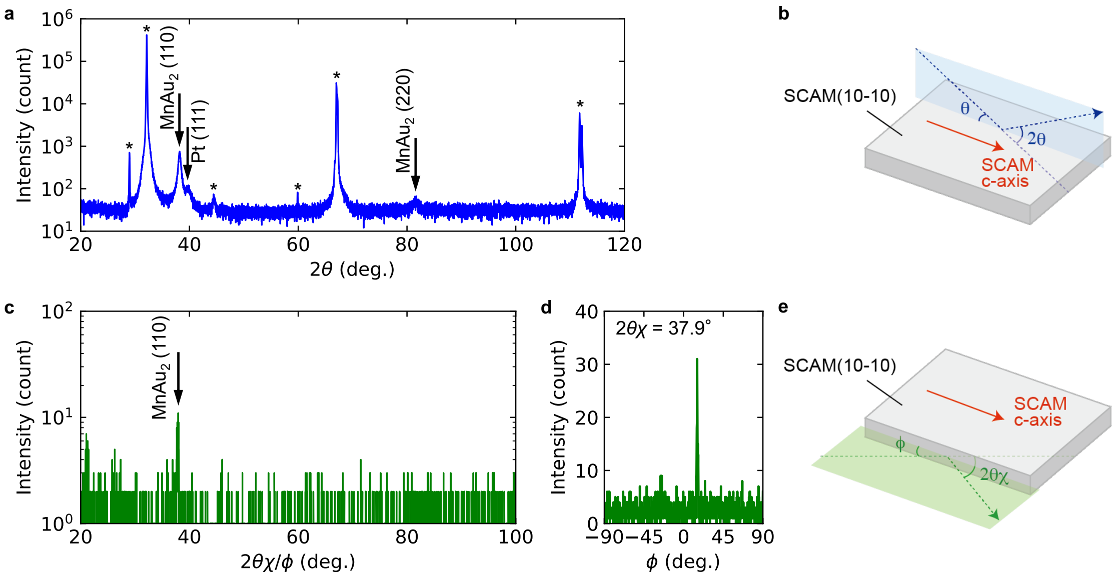

Supplementary Fig. 4 | XRD profiles of the $MnAu_2$ / Pt bilayer film sample.

a Out-of-plane $\theta/2\theta$-scan. $MnAu_2$ (110) and (220) reflections indicate that the $MnAu_2$ (110) plane is grown on the $ScMgAlO_4$ (10−10) plane. Pt (111) reflection indicates the orientation of the Pt (111) plane. Asterisks denote the peaks from the $ScMgAlO_4$ (SCAM) substrate or sample stage. No extra reflections were observed.

b Schematic illustration of the out-of-plane $\theta/2\theta$-scan.

c In-plane $2\theta\chi/\varphi$-scan. The $MnAu_2$ (110) reflection indicates that the $MnAu_2$ [1−10] direction is perpendicular to the $ScMgAlO_4$ [0001] direction.

d In-plane φ-scan. A single sharp peak in the φ-scan range from −90° to 90° indicates the in-plane single-crystal order of the $MnAu_2$ layer.

e Schematic illustration of the in-plane $2\theta\chi/\varphi$- and φ- scans.

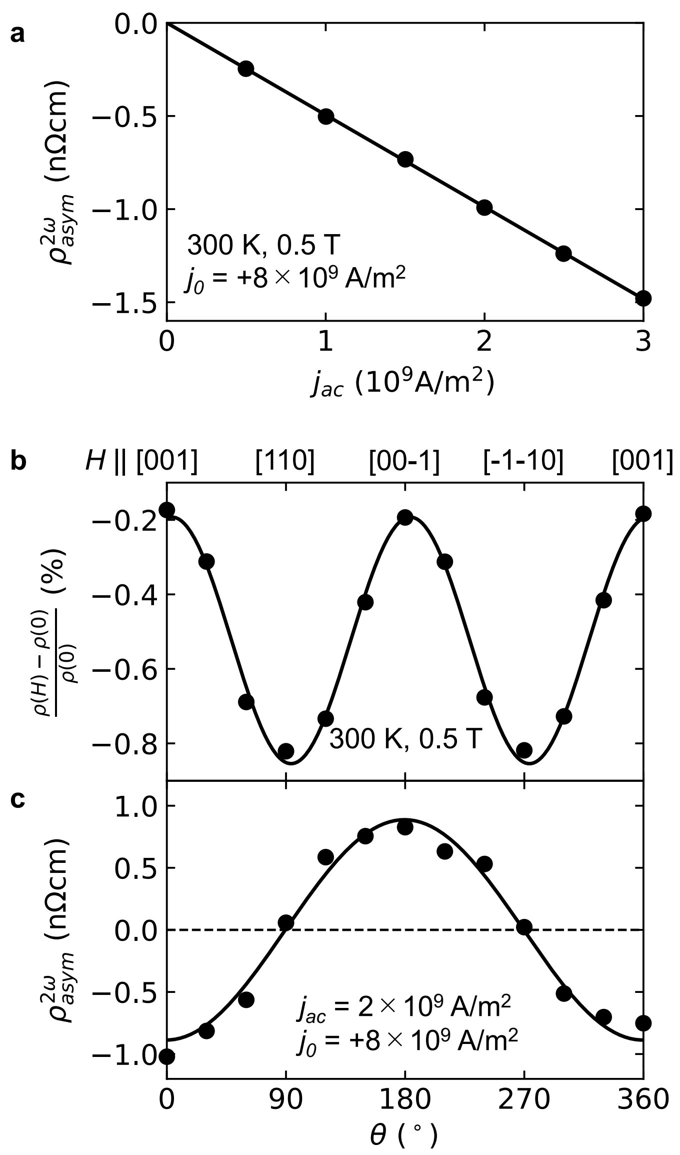

Supplementary Fig. 5 | Properties of nonreciprocal electronic transport in $MnAu_2$.

a The ac current j_{ac} dependence of $\rho^{2\omega}_{asym}$ at 300 K and 0.5 T after the field sweep from H_0 = +3 T with j_0 = +8.0×10^9 A/m^2. $\rho^{2\omega}_{asym}$ shows a linear j_{ac} dependence, being consistent with the picture of nonreciprocal electric transport.

b Magnetoresistance $[\rho(H) - \rho(0)]/\rho(0)$ as a function of magnetic field angle θ at 300 K and 0.5 T. Here θ is the angle between the current and the magnetic field. The magnetic field is rotated within the (1−10) plane. The solid line is the result of fitting to cos2θ. The 180-degree rotation of the magnetic field does not alter the linear resistivity.

c θ dependence of $\rho^{2\omega}_{asym}$ at 300 K and 0.5 T after the field sweep from H_0 = +3 T with j_0 = +8.0×10^9 A/m^2. $\rho^{2\omega}_{asym}$ shows cosθ angle dependence, and the magnitude is maximum at θ = 0 and 180 deg., being consistent with the chiral symmetry.

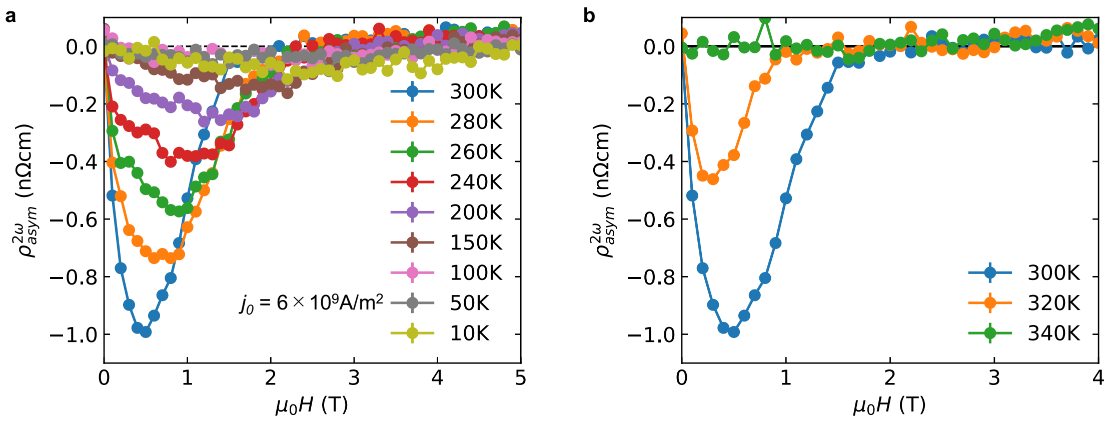

Supplementary Fig. 6 | Field sweep chirality control at various temperatures in $MnAu_2$.

a, b Magnetic field dependence of $\rho^{2\omega}{}_{asym}(H)$ after the field sweep from H_0 = +5 T with j_0 = +6.0×10^9 A/m^2 at various temperatures. The field sweep control and the measurement of $\rho^{2\omega}{}_{asym}(H)$ are performed at the same temperature.

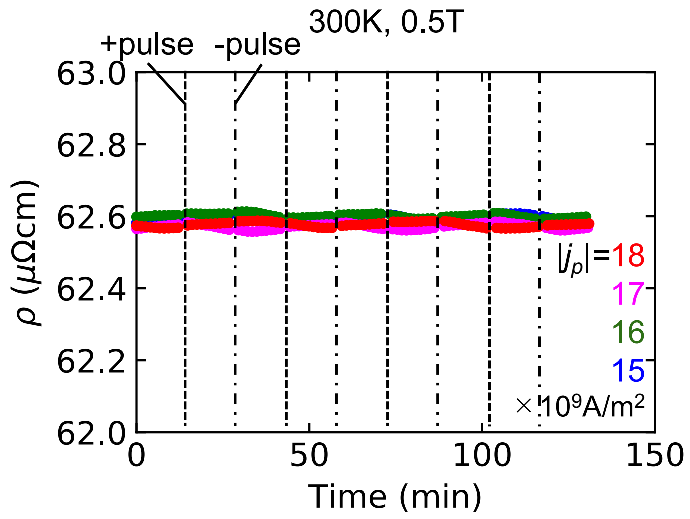

Supplementary Fig. 7 | First-harmonic resistivity during the electric current pulses application.

Temporal variation of first harmonic resistivity ρ during the measurement for Fig. 4a in the main text. The variation in ρ during the switching sequence is less than 0.1 %, indicating negligible sample damage due to the switching pulses.

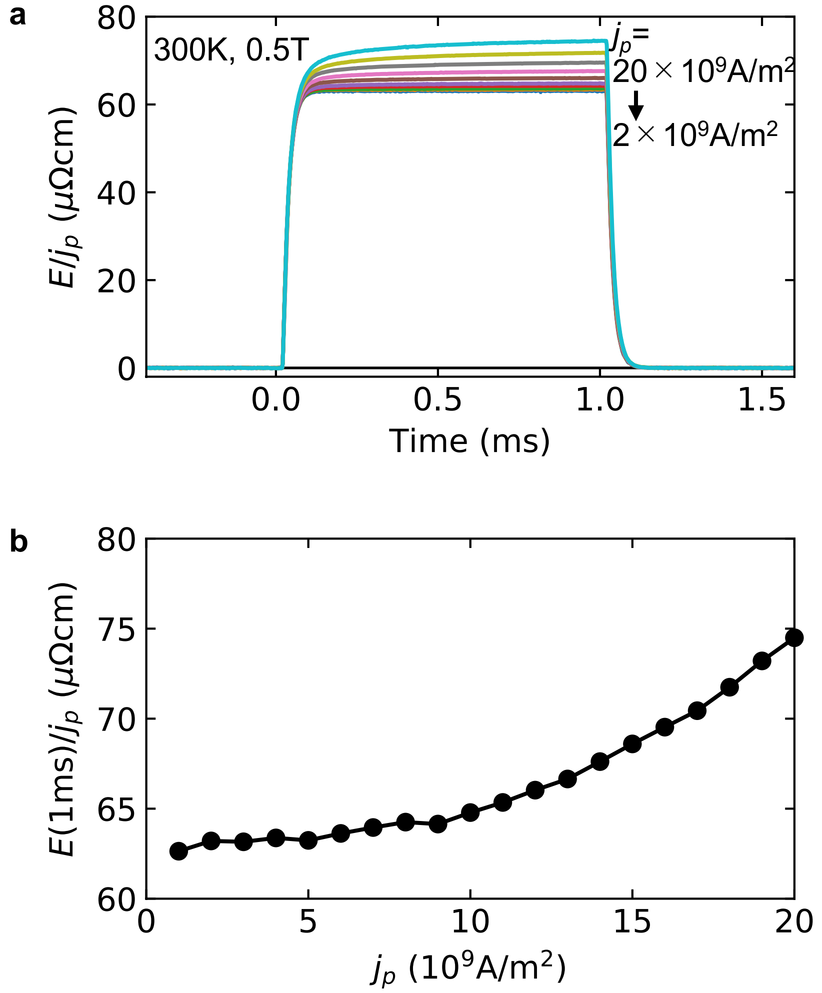

Supplementary Fig. 8 | Estimation of sample heating by the application of electric current pulse.

a Time dependence of the resistivity E/j_p during the current pulse application with various magnitudes for the chirality switching measurement (Fig. 4 in the main text).

b j_p dependence of resistivity E/j_p. In this figure, E/j_p at 1 ms is adopted. We estimated the sample temperature during the pulse application from these data and the temperature dependence of resistivity shown in Fig. 2d in the main text.

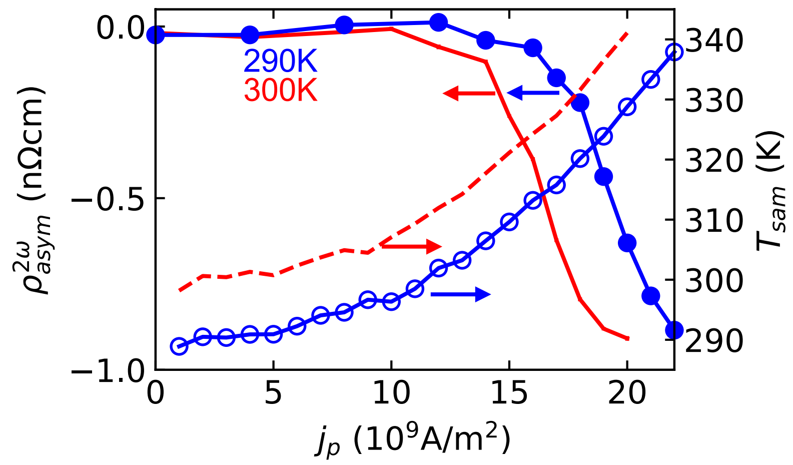

Supplementary Fig. 9 | Comparison of chirality switching results at 290 K and 300 K.

The j_p dependence of $\rho^{2\omega}{}_{asym}$ and the sample temperature T_{sam} during the application of current pulse at nominal experimental temperatures 290 K and 300 K for the chirality switching phenomena (Fig.4 in the main text). The experimental probe was controlled to the nominal temperature, but the sample was heated by the electric current pulse. When the nominal temperature is decreased to 290 K, the j_p dependences are horizontally shifted, indicating that the sample temperature is important for the j_p dependence.

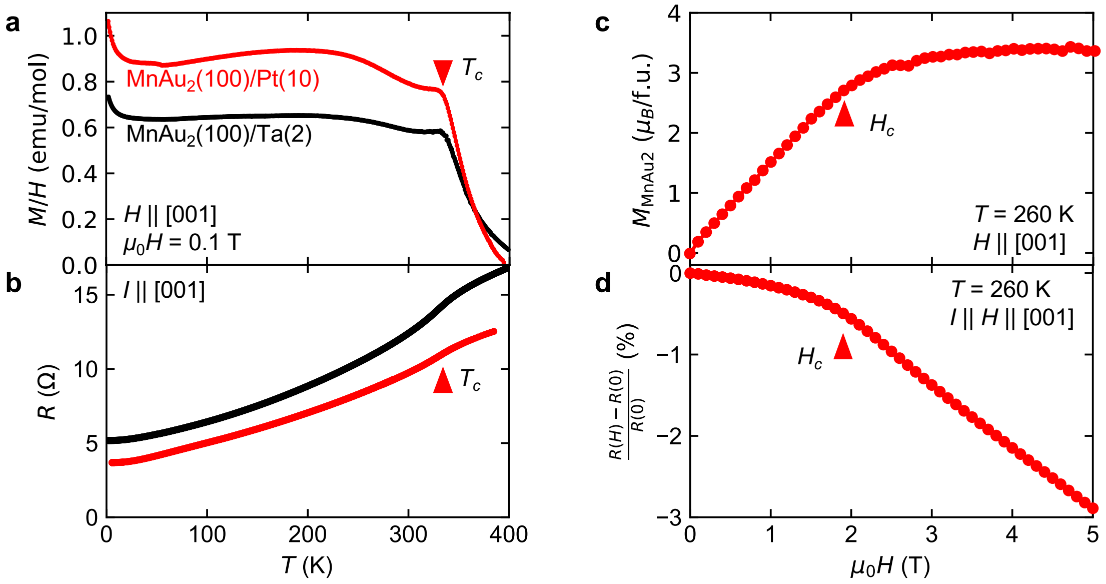

Supplementary Fig. 10 | Properties of the $MnAu_2$ / Pt bilayer film sample.

a Temperature, T, dependence of the magnetic susceptibility M/H of the $MnAu_2$ / Pt sample, which is obtained by the magnetization M divided by the magnetic field H as large as 0.1 T. The data for the $MnAu_2$ (100nm) / Ta (2 nm) sample are reproduced from Fig. 2c in the main text. The triangle denotes the helimagnetic transition temperature T_c = 335 K.

b T dependence of the longitudinal resistance R for the $MnAu_2$ / Pt sample. The data for the $MnAu_2$ (100nm) / Ta (2 nm) sample are reproduced from Fig. 2d in the main text.

c Magnetic field H dependence of the magnetization M_{MnAu2} at 260 K for the $MnAu_2$ / Pt sample. The linear diamagnetic contribution is subtracted from the measured magnetization. The triangle denotes the helimagnetic to ferromagnetic transition field H_c.

d H dependence of the longitudinal magnetoresistance $(R(H) - R(0)) / R(0)$ at 260 K for the $MnAu_2$ / Pt sample. The triangle denotes the helimagnetic to ferromagnetic transition field H_c.

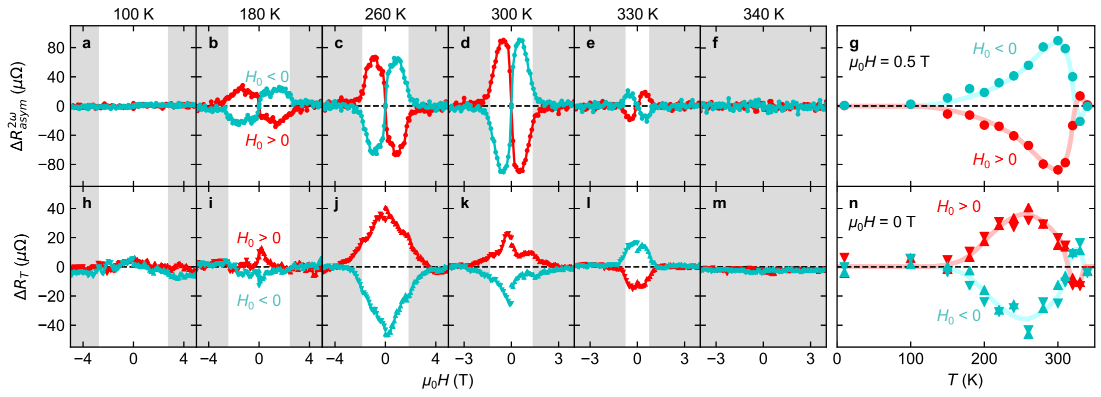

Supplementary Fig. 11 | Temperature dependence of $\rho^{2\omega}{}_{asym}(H)$ and the chirality-dependent transverse resistance ΔR_T in the $MnAu_2$ / Pt bilayer film sample.

a-f Magnetic field dependence of the averaged NET signal $\Delta R^{2\omega}{}_{asym} = (R^{2\omega}{}_{asym}(+I_0) - R^{2\omega}{}_{asym}(-I_0))/2$ at various temperatures. Here, the chirality control procedure and the measurements were performed at the same temperature. Red and cyan symbols show $\Delta R^{2\omega}{}_{asym}$ for $H_0 > 0$ and $H_0 < 0$, respectively. While the data of $H < 0$ are merely copies of the $H > 0$ data, we plot the $H < 0$ data just for clarity. The gray shading represents the induced-FM or paramagnetic phases.

g Temperature, T, dependence of $\Delta R^{2\omega}{}_{asym}$ at 0.5 T for $H_0 > 0$ (red) and $H_0 < 0$ (cyan). Solid lines are guides to the eyes. $\Delta R^{2\omega}{}_{asym}$ is observed in the helimagnetic state below T_c = 335 K, and the sign of $\Delta R^{2\omega}{}_{asym}$ at 330K is opposite to that at 300 K (see also Supplementary Fig. 12).

h-m Magnetic field dependence of ΔR_T at various temperatures. Red and cyan symbols show ΔR_T for $H_0 > 0$ and $H_0 < 0$, respectively.

n T dependence of ΔR_T at 0 T for $H_0 > 0$ (red) and $H_0 < 0$ (cyan). Solid lines are guides to the eyes. ΔR_T gradually evolves below T_c, shows a sign reversal just below the transition temperature, and the magnitude almost vanishes below 100 K. The temperature dependence of ΔR_T is surprisingly similar to that of $\Delta R^{2\omega}{}_{asym}$; both show sign changes, the magnitudes show a maximum at 260–300 K, and both vanish around 100 K. The similarity implies that the mechanisms of these two phenomena are microscopically related, although the details remain to be clarified.

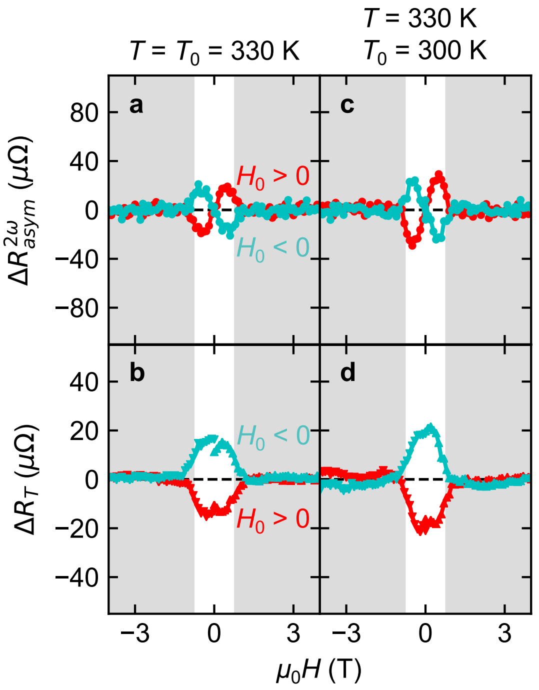

Supplementary Figure 12 | Comparison of nonreciprocal electrical transports and transverse resistances after chirality control at 300 K and 330 K.

a, b Magnetic field H dependence of $\Delta R^{2\omega}{}_{asym}$ (**a**) and ΔR_T (**b**) at 330 K for the chirality control temperature T_0 = 330 K reproduced from Supplementary Figs. 11e and 11l.

c, d Magnetic field H dependence of $\Delta R^{2\omega}{}_{asym}$ (**c**) and ΔR_T (**d**) at 330 K for the chirality control temperature T_0 = 300 K. The signs were the same as those for T_0 = 330 K shown in **a** and **b**, which indicates that the sign changes of $\Delta R^{2\omega}{}_{asym}$ and ΔR_T are not induced by the reversal of controlled chirality.

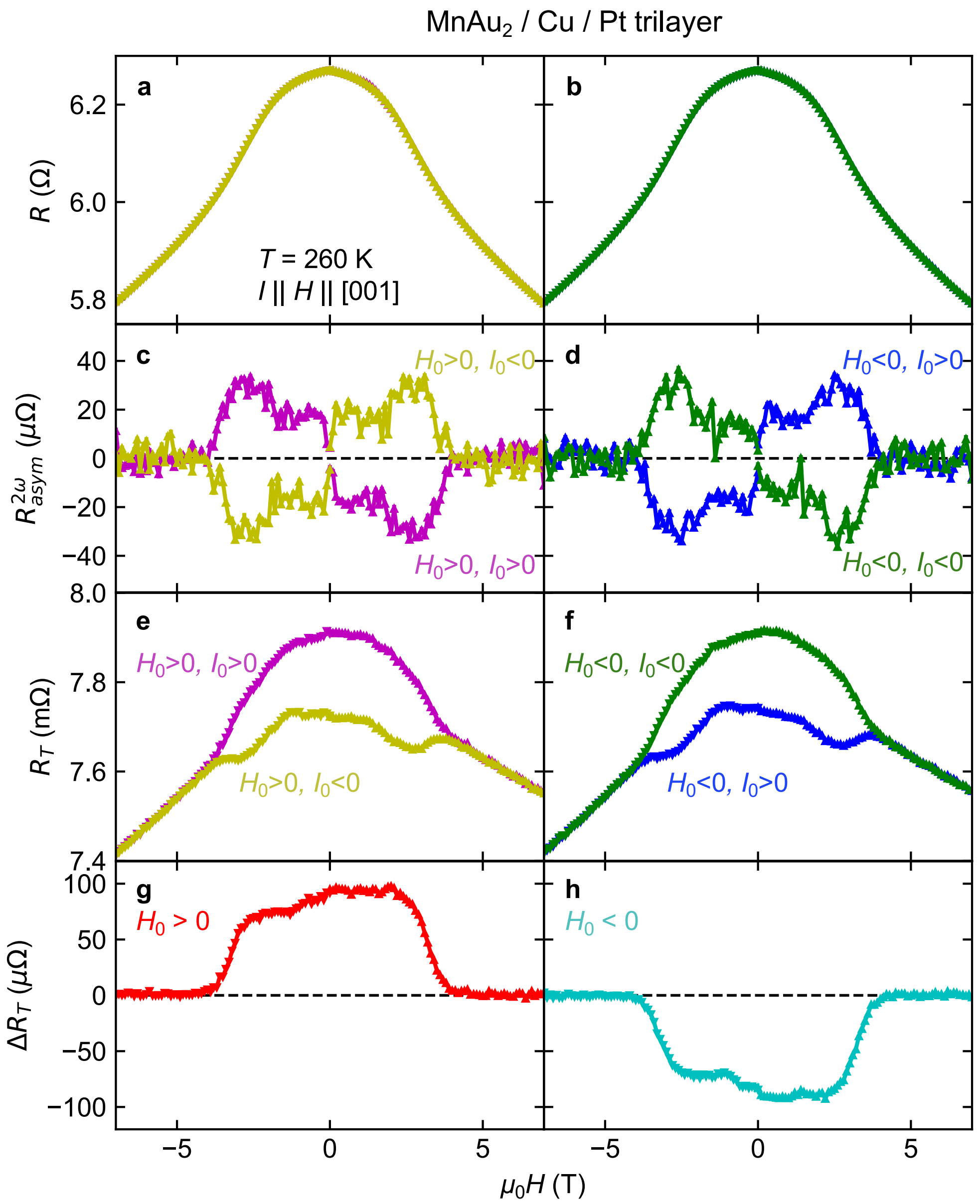

Supplementary Figure 13 | Nonreciprocal electric transport and chirality-dependent transverse resistance in the MnAu₂ / Cu / Pt trilayer film sample.

a, b Longitudinal resistance R, **c, d** nonreciprocal 2nd harmonic resistance $R^{2\omega}{}_{asym}$, **e, f** transverse resistance R_T, and **g, h** the chirality-dependent component $\Delta R_T = (R_T(+I_0) - R_T(-I_0))/2$ at 260 K for a $MnAu_2$ 100 nm / Cu 1 nm / Pt 10 nm trilayer film sample. Before the measurements, the magnetic field H_0 was swept from ±7 T to ±3 T traversing the transition magnetic field with the application of dc electric current I_0 = ±8.0 mA to control the chirality. The measurements were performed with an ac current amplitude of 1 mA.

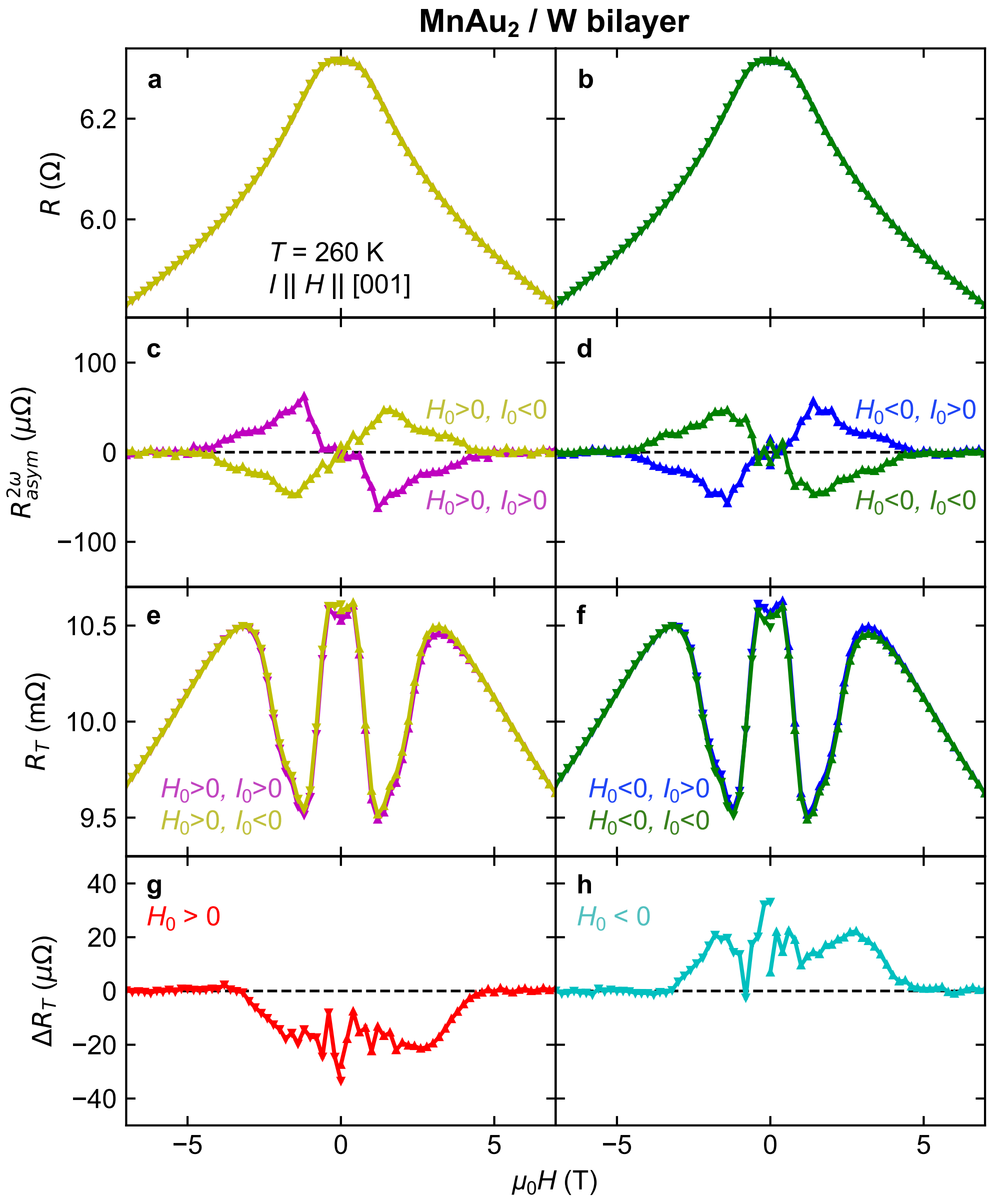

Supplementary Figure 14 | Nonreciprocal electric transport and chirality-dependent transverse resistance in the $MnAu_2$ / W bilayer film sample.

a, b Longitudinal resistance R, **c, d** nonreciprocal 2nd harmonic resistance $R^{2\omega}_{asym}$, **e, f** transverse resistance R_T, and **g, h** the chirality-dependent component $\Delta R_T = (R_T(+I_0) - R_T(-I_0))/2$ at 260 K for a $MnAu_2$ 100 nm / W 10 nm bilayer film sample. Before the measurements, the magnetic field H_0 was swept from ±7 T to ±3 T traversing the transition magnetic field with the application of dc electric current I_0 = ±8.0 mA to control the chirality. The sign of ΔR_T is opposite to that of the $MnAu_2$ / Pt bilayer device, reflecting the opposite signs of spin Hall conductance in Pt and W[7].

Supplementary References

1. Watanabe, H., Hoshi, K. & Ohe, J. Chirality-induced spin current through spiral magnets. *Phys. Rev. B* **94**, 125143 (2016).
2. Jiang, N., Nii, Y., Arisawa, H., Saitoh, E. & Onose, Y. Electric current control of spin helicity in an itinerant helimagnet. *Nat. Commun.* **11**, 1601 (2020).
3. Ohe, J. I. & Onose, Y. Chirality control of the spin structure in monoaxial helimagnets by charge current. *Appl. Phys. Lett.* **118**, (2021).
4. Ando, T. Quantum point contacts in magnetic fields. *Phys. Rev. B* **44,** 8017 (1991).
5. Nagamiya, T. Helical Spin Ordering—1 Theory of Helical Spin Configurations. *Solid State Physics* **20,** 305 (1968).
6. Rikken, G. L. J. A., Fölling, J., & Wyder, P., *Phys. Rev. Lett* **87,** 236602 (2001).
7. Sinova, J., Valenzuela, S. O., Wunderlich, J., Back, C. H. & Jungwirth, T. Spin Hall effects. *Rev. Mod. Phys.* **87**, 1213-1260 (2015).
